# Comprehensive Family Caregiver Support and Caregiver Well-Being: Preliminary Evidence From a Pre-post-survey Study With a Non-equivalent Control Group

**DOI:** 10.3389/fpubh.2019.00122

**Published:** 2019-05-22

**Authors:** Valerie A. Smith, Jennifer Lindquist, Katherine E. M. Miller, Megan Shepherd-Banigan, Maren Olsen, Margaret Campbell-Kotler, Jennifer Henius, Margaret Kabat, Courtney Harold Van Houtven

**Affiliations:** ^1^Department of Population Health Sciences, Duke University, Durham, NC, United States; ^2^Health Services Research and Development, Durham VA Health Care System, Durham, NC, United States; ^3^Division of General Internal Medicine, Department of Medicine, Duke University, Durham, NC, United States; ^4^Health Policy and Management, University of North Carolina, Chapel Hill, NC, United States; ^5^Department of Biostatistics and Bioinformatics, Duke University, Durham, NC, United States; ^6^Caregiver Support Program, United States Department of Veterans Affairs, Seattle, WA, United States

**Keywords:** informal caregiver, informal care, policy intervention, pre-post-survey, caregiver well-being, depressive symptoms, quality of life, veterans

## Abstract

**Introduction:** In May 2010, the Caregivers and Veterans Omnibus Health Services Act of 2010, was signed into law in the United States, establishing the Program of Comprehensive Assistance for Family Caregivers (PCAFC) provided through the VA Caregiver Support Program (CSP). Prior to this program, over half of family caregivers reported being untrained for the tasks they needed to provide. The training through PCAFC represents the largest effort to train family caregivers in the U.S., and the features of the program, specifically a monthly stipend to caregivers and access to a Caregiver Support Coordinator at each VA medical center nationally, make it the most comprehensive caregiver support program ever enacted in the U.S.

**Methods:** The purpose of this study is to examine the association between PCAFC participation and caregiver well-being following enrollment, comparing participating PCAFC caregivers to caregivers who applied to but were not approved for PCAFC participation (non-participants). Well-being is defined using three diverse but related outcomes: depressive symptoms, perceived financial strain, and perceived quality of the Veteran's health care. Additional well-being measures also examined include the Zarit Burden Inventory and positive aspects of caregiving.

**Results:** The survey sample comprised of 92 caregivers approved for PCAFC and 66 caregivers not approved. The mean age of responding caregivers was 45; over 90% of caregivers were female; and over 80% of caregivers were married in both groups. We find promising trends in well-being associated with PCAFC participation. First, the perception of financial strain declined among participants compared to non-participants. Second, while depressive symptoms did not improve for the PCAFC caregivers, depressive symptoms increased among non-participants. Third, perceived quality of the Veteran's VA healthcare was no different between participants and non-participants. However, the 158 returned surveys reflect only a 5% response rate; hence this evidence is preliminary.

**Conclusion:** Despite cautioning that results be interpreted as preliminary, this study provides unique descriptive information about young caregivers of U.S. post-9/11 Veterans, and offers a first step in filling the evidence gap about how comprehensive caregiver support in the U.S. may affect caregiver well-being. These preliminary findings should be explored and validated in a larger sample.

## Introduction

In 2014 the RAND Corporation released a report entitled “Hidden Heroes: America's Military Caregivers” (1). The report described the military caregiver population, the current formalized, public support offered to caregivers, and offered recommendations to improve the well-being of military caregivers. The survey found significant demographic differences between post-9/11 military caregivers and civilian caregivers as well as differences in utilization of caregiving services, social support, and the nature of caregiving tasks. For example, fewer post-9/11 military caregivers reported having a support system compared to civilian caregivers. Post-9/11 military caregivers helped with fewer Activities of Daily Living and Instrumental Activities of Daily Living than civilian caregivers, but provided greater assistance in helping “care recipients cope with stressful situations or avoid triggers of anxiety or antisocial behavior.” The report also highlighted the burden of caregiving on pre- and post-9/11 military caregivers, including work strain, financial strain and difficulty planning for the future. Post-9/11 military caregivers, estimated to number 1.1 million, are often younger than the caregiver populations that have been studied previously, as are their care recipients. In a non-military population, there is evidence that caregivers aged 18–25 also identified a variety of unmet needs, including difficulty obtaining medical help and information (2).

Whereas, the evidence is clear that providing informal care can cause adverse emotional and physical health effects on elderly spousal caregivers and adult children caring for elderly parents (3, 4), the long-term health effects of caregiving for traumatically injured younger Veterans is not well-understood. We know from recent studies that caregivers of Veterans with polytrauma and TBI provide intensive and varied care, and that many experience financial strain in the short-term, especially intensive caregivers, defined as those providing more than 20 h of care a week (5). Exits from the labor force occurred among 40% of military caregivers, and caregivers commonly reported accumulating debt and depleting assets over time (6). In this same study population, there is evidence that Veterans' neurobehavioral problems and intensity of required care were associated with higher caregiver burden, and higher burden was associated with poor caregiver mental health (7–9). Caregivers also reported feeling unprepared; for example, only approximately half of caregivers of Veterans with TBI and Polytrauma reported that they had received training about how to help their Veteran (10). Furthermore, there is evidence that caregivers of Veterans with polytrauma, including TBI, who have not received training experienced higher anxiety, depression, caregiver burden, and lower self-esteem than those who received training (10). These studies used cross-sectional data and are from the mid-2000s, so could not address changes over time in well-being and mental health outcomes. Thus, understanding the short-, medium-, and long-term effects of caregiving on post-9/11 military caregivers in the current policy climate is crucial in order to mitigate future negative outcomes.

Responding to calls from Veteran Service Organizations, families, and other stakeholders for more systematic supports to meet the demonstrated training and financial needs of caregivers of post-9/11 Veterans, in May 2010, PL 111-163, the Caregivers and Veterans Omnibus Health Services Act of 2010, was signed into law by congress. The law established the Program of Comprehensive Assistance for Family Caregivers (PCAFC), which has specific eligibility requirements for the Veteran and the family caregiver and provides a series of services and supports for the family caregiver, provided through the VA Caregiver Support Program (CSP). These supports include a monthly stipend paid directly to the family caregiver, access to health care if not already covered under a health insurance plan, education and training, travel, lodging and subsistence, respite care, and mental health services (see Van Houtven et al 2017 for more detail on the program components). Specifically, it is available to qualifying caregivers who care for Veterans injured in the line of duty on or after 9/11/2001. Prior to the implementation of VA's Caregiver Support Program generally, and PCAFC specifically, caregiver training, if provided, was generally delivered in a clinical setting, was *ad hoc*, and based on interactions between caregivers and individual providers. The caregiver training provided by PCAFC since 2011 represents the largest effort to train family caregivers in the U.S.; as of June 1, 2017, 37,597 caregivers had completed the required 9-part training program. Combined with the other features, including the stipend (averages $600–$2,300 a month depending on acuity of the patient and the caregiving intensity), optional health insurance for caregivers, and short-term respite care, PCAFC is the most comprehensive caregiver support program ever enacted in the U.S. Because PCAFC provides an array of supports simultaneously, it is not possible to disentangle the mechanisms by which the program components affect individual caregiver well-being. There is some evidence that although optional supports are highly valued by caregivers, such as direct counseling from Caregiver Support Coordinators at each medical center, they are not commonly used (11). Therefore, given that all caregivers received required caregiver training and the monthly tax free financial stipend, these two features may serve as the primary mechanisms for affecting caregiver well-being.

The purpose of this study is to examine the association between PCAFC participation and caregiver well-being over 9 months, comparing participating PCAFC caregivers to caregivers who applied to but were not approved for PCAFC participation (non-participants). In this paper, well-being encompasses depressive symptoms, perceived financial strain, and perceived quality of the Veteran's recent health care in the VA health care system (the VA health care system is a public integrated health care system serving nearly 9 million Veterans in the U.S.). The data are derived from caregivers who responded to a national survey at both of two time points in 2015. Because we received only a 5% response rate, the results should be interpreted as preliminary findings that will need to be explored and validated in a larger sample. This study is a first step in filling the evidence gap about how comprehensive caregiver support in the U.S. may affect caregiver well-being and provides descriptive information on well-being among a young, under studied group of caregivers—family caregivers of post-9/11 Veterans with significant care needs.

## Materials and Methods

### Study Sample

This survey study was designed using a pre-post-design with a non-equivalent control group.

Caregivers who had applied to PCAFC and whose applications were in process of evaluation as of December 8, 2014 (*n* = 3,401) were mailed a survey to complete and return by mail in a large, stamped, addressed envelope (Survey 1). After ~9 months, caregivers who were mailed Survey 1, regardless of their response to that survey, were mailed an invitation to complete the same survey in a web-based format (Survey 2). This timeframe was chosen to allow caregivers who were in process of applying for PCAFC at the time of Survey 1 to have been approved and enrolled for long enough to have received a sufficient “dose” of PCAFC, or to have been denied. The administrators of the program (The VA Caregivers Support Program or CSP) provided a list of approved and denied caregivers, along with approval/denial dates, resulting in respondents being classified as participating, denied, or still in-process as of September 1, 2015. Criteria for inclusion in the analytical data set included: responding to both Survey 1 and Survey 2; never having been previously approved for PCAFC by the time Survey 1 was fielded; having an application still in-process when Survey 1 was completed; and, for those respondents classified as participating by the time of Survey 2 survey, having been enrolled in PCAFC for at least 90 days as of September 1, 2015. The individuals classified as denied or still in-process at the time of Survey 2 were collapsed into a single group, “non-participants,” and served as the non-equivalent control group in the analysis. Recipients of Survey 1 received a pen in their mailed survey packet as an incentive for completing the survey, but budget limitations precluded recipients of Survey 2 from receiving any tokens of appreciation. No recipient received a financial incentive for completing the survey.

### Well-Being Outcomes

The outcomes of interest were the change in response between Survey 1 and Survey 2 for each of the measures, comparing participating caregivers with non-participating caregivers. The primary outcomes were defined as the change in the scores (Survey 2 minus Survey 1) for the following variables: financial strain; depressive symptoms; and rating of quality of VA health care received (12). Secondary outcomes were defined as caregiver burden and positive aspects of caregiving.

#### Caregiver Perceived Financial Strain (13)

We measured perceived financial strain through the three-item Impact on Finances subscale from the Caregiver Reaction Assessment. Each item was scored on a 1–5 scale, and the strain score was the mean of the scores for the 3 items (14, 15); higher scores indicate a higher level of strain. The change in the score is the difference of Survey 2 minus Survey 1, creating a possible change in score from −4 to 4, with a negative value indicating a reduction in strain. The scale asks caregivers to state the degree to which they agree: “It is difficult to pay for the things the Veteran needs”; “Caring for the Veteran puts a financial strain on me”; and, “My financial resources are adequate to pay for things that are required for caregiving.” Response options include “Strongly Disagree,” “Disagree,” “Neither Agree nor Disagree,” “Agree,” or “Strongly Agree.”

#### Caregiver Depressive Symptoms (16)

We measured caregiver depressive symptoms through the Center for Epidemiologic Studies Depression 10-item Scale (CESD-10). The score is a sum of 10 items with response 0–3, thus scores range from 0 to 30, where higher scores indicate more depressive symptoms. The change in CESD-10 resulted in a possible range of −30 to 30, with a negative value indicating a decrease in depressive symptoms.

#### Caregiver's Global Satisfaction With Veterans Health Administration (VHA) Care (17)

We measured caregiver's global rating of satisfaction with the Veteran's VHA care through a single item from the Consumer Assessment of Healthcare Providers and Systems (CAHPS) 2013 Health Plan survey. “Using any number from 0 to 10, where 0 is the worst health care possible and 10 is the best health care possible, what number would you use to rate all the health care the Veteran received at the VA?” Scores range from 0 to 10, where higher scores indicate better care. The difference in response can range from −10 to 10, with a positive value indicating an increase in quality rating.

#### Caregiver Burden

Caregiver burden was defined using the 12-item Zarit burden measure, described as the level of stress felt by a caregiver (18, 19). The scale covers factors most often mentioned by caregivers as problems, including health, psychological well-being, finances, social life, and the relationship shared by the caregiver and care recipient. Caregiver programs have been shown to improve caregiver burden modestly (20, 21). The Zarit scale can range from 0 to 48, with a higher value indicating more burden/stress. A score >16 suggests clinically significant caregiver burden (18, 22). The difference in response can range from −48 to 48, with a negative value indicating a decrease in burden/stress.

#### Positive Aspects of Caregiving

We examined the positive aspects of caregiving measure, a reliable and well-validated measure developed by Tarlow et al. (23). This score ranges from 9 to 45 and a higher score indicates perception of more positive aspects of caregiving. The difference in response can range from −36 to 36, with a positive value indicating an increase in quality rating.

### Statistical Analyses

All caregiver well-being outcomes were evaluated descriptively for trends over time within participants and non-participants. Initially the statistical plan for the primary outcomes was conceived as a propensity-score weighted comparative effectiveness design to obtain national estimates of the impact of PCAFC on caregiver well-being. Due to the low overall response rate and high levels of missingness in baseline covariate values, we were unable to estimate covariate-adjusted models.

Therefore, we calculated simple *t*-tests on the well-being change scores between surveys, in an effort to control for baseline differences in well-being. All analyses were conducted in SAS 9.4 (SAS Institute, Cary, NC) and statistical significance levels were set a priori at 0.05. The results should be interpreted as preliminary findings that will need to be explored and validated in a larger sample. As a VA quality improvement project, this work was not subject to institutional review board approval in accordance with the local legislation and institutional requirements.

## Results

### Final Analytic Cohort

**Figure 1:**
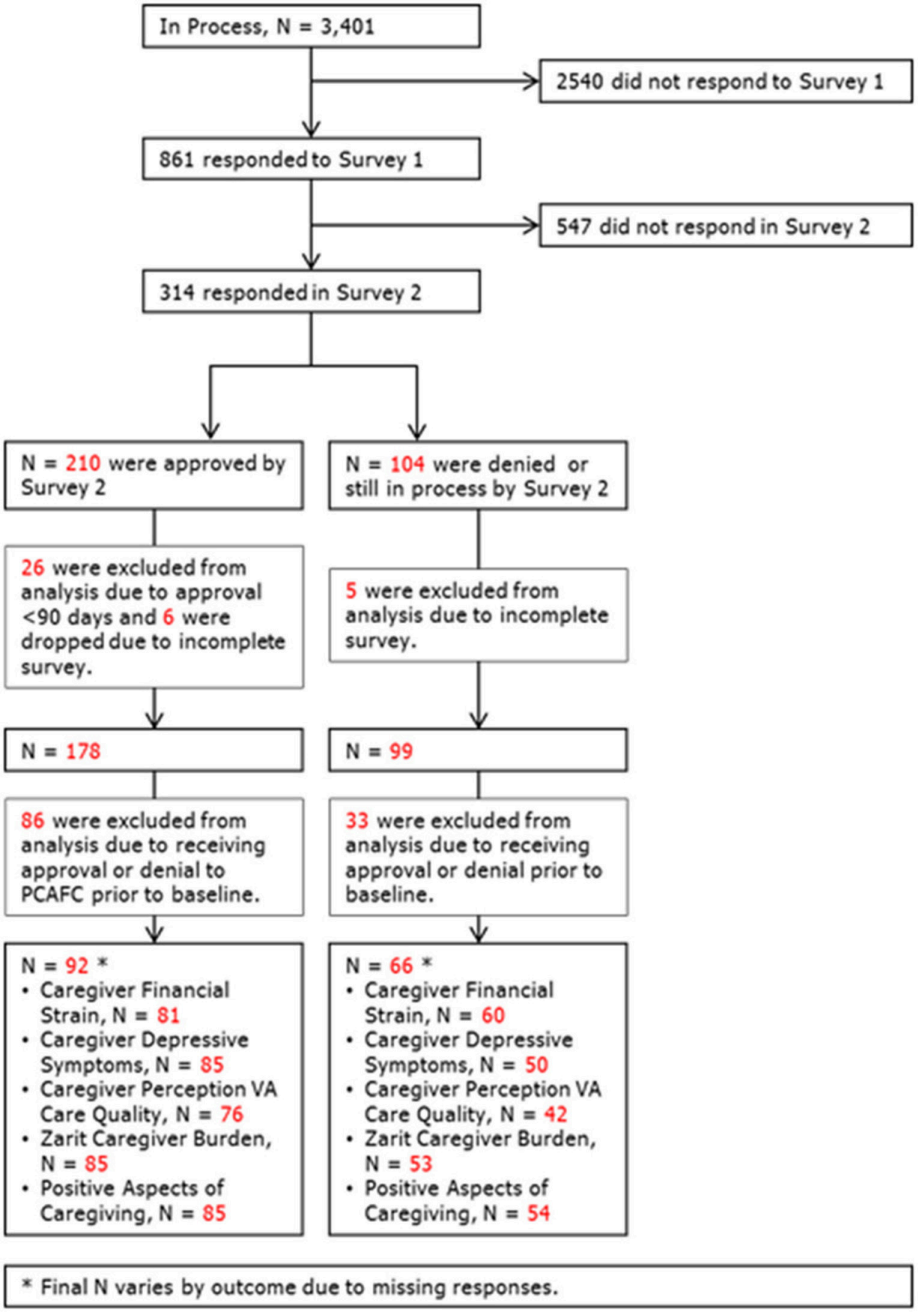
Final analytical cohort. After applying our inclusion and exclusion criteria, the resulting dataset used for this study consisted of 92 approved caregivers (participants) and 66 not approved caregivers (non-participants).The total sample size was 158 respondents.

After applying the inclusion criteria, the resulting dataset consisted of 92 caregivers approved for PCAFC (participants) and 66 caregivers not approved (non-participants) (Figure 1). The resulting sample size, 158 respondents, is <5% of the original cohort of 3,401 who were invited to participate in the survey. Results, while informative about potential trends, may not generalize to or represent the experience of all caregivers participating in PCAFC, and therefore should be considered preliminary.

### Descriptive Results

#### Characteristics of the Study Cohort

Table 1 displays the descriptive statistics for the analytical cohort. The average age of caregivers who responded to both surveys was ~45; over 90% of caregivers were female; and over 80% of caregivers were married in both groups. There were descriptively similar numbers of White, Black or African American, and Hispanic respondents in the participant and non-participant groups. One-tenth of the caregivers in the participant group were Veterans themselves, compared to just under one-twentieth of the caregivers in the non-participant group. Caregivers participating in the PCAFC had been caregiving similar amounts of time as non-participating caregivers (4.65 years compared to 4.92 years, respectively). Caregivers in both groups were primarily spouses or significant others (participants = 86% and non-participants = 82%).

**Table 1:** Baseline caregiver demographics.

	**Analytical Cohort**			
	**Participants**, ***N*** **=** **92**		**Non-participants**, ***N*** **=** **66**	
**Gender, % (*****n*****)**	0%	(*n* = 0)	3.03%	(*n* = 2)
Missing				
Male	8.70%	(*n* = 8)	3.03%	(*n* = 2)
Female	91.30%	(*n* = 84)	93.94%	(*n* = 62)
**Age**, **mean (*****SD*****)**	44.29 (12.19)	(*n* = 92)	44.62 (11.57)	(*n* = 66)
**Marital status, % (*****n*****)**	80.43%	(*n* = 74)	86.36%	(*n* = 57)
Missing				
Living together, committed relationship	10.87%	(*n* = 10)	6.06%	(*n* = 4)
Divorced/Separated	5.43%	(*n* = 5)	3.03%	(*n* = 2)
Widowed	1.09%	(*n* = 1)	1.52%	(*n* = 1)
Single, never married	2.17%	(*n* = 2)	3.03%	(*n* = 2)
**Race, % (*****n*****)**	5.43%	(*n* = 5)	9.09%	(*n* = 6)
Missing				
White	56.52%	(*n* = 52)	53.03%	(*n* = 35)
Black or African American	31.52%	(*n* = 29)	30.30%	(*n* = 20)
Other	2.17%	(*n* = 2)	1.52%	(*n* = 1)
Multiple Races	4.35%	(*n* = 4)	6.06%	(*n* = 4)
**Ethnicity, % (*****n*****)**	1.09%	(*n* = 1)	3.03%	(*n* = 2)
Missing				
No, not Hispanic	82.61%	(*n* = 76)	80.30%	(*n* = 53)
Yes, Hispanic	16.30%	(*n* = 15)	16.67%	(*n* = 11)
**Veteran status, % (*****n*****)**	0.0%	(*n* = 0)	1.52%	(*n* = 1)
Missing				
Caregiver is not a Veteran	89.13%	(*n* = 82)	93.94%	(*n* = 62)
Caregiver is a Veteran	10.87%	(*n* = 10)	4.55%	(*n* = 3)
**Length of time (years) as a caregiver, mean (*****SD*****)**	4.65 (3.55)	(*n* = 80)	4.92 (6.88)	(*n* = 56)
**Number of days per week, mean (*****SD*****)**	4.84 (0.54)	(*n* = 89)	4.70 (0.99)	(*n* = 64)
**Number of days per weekend, mean (*****SD*****)**	1.92 (0.27)	(*n* = 89)	1.80 (0.48)	(*n* = 64)
**Relationship to veteran % (*****n*****)**	1.09%	(*n* = 1)	3.03%	(*n* = 2)
Missing				
Spouse or Significant other	85.87%	(*n* = 79)	81.82%	(*n* = 54)
Parent	8.70%	(*n* = 8)	12.12%	(*n* = 8)
Child	0%	(*n* = 0)	3.04%	(*n* = 2)
Sibling	1.09%	(*n* = 1)	0%	(*n* = 0)
Other	3.26%	(*n* = 3)	0%	(*n* = 0)
**Living situation relative to veteran, % (*****n*****)**	0.0%	(*n* = 0)	3.03%	(*n* = 2)
Missing				
In the same house	95.65%	(*n* = 88)	89.39%	(*n* = 59)
Within walking distance	1.09%	(*n* = 1)	0%	(*n* = 0)
Within 20-min of driving distance from my home	2.17%	(*n* = 2)	3.03%	(*n* = 2)
Between 20 min and an hour of driving distance from my home	0%	(*n* = 0)	4.55%	(*n* = 3)
Over an hour of driving distance from my home	1.09%	(*n* = 1)	0%	(*n* = 0)
**Education level, % (*****n*****)**	2.17%	(*n* = 2)	1.52%	(*n* = 1)
Missing				
Less than high school	5.43%	(*n* = 5)	4.55%	(*n* = 3)
Completed high school or GED	19.57%	(*n* = 18)	13.64	(*n* = 9)
Completed trade/technical school	3.26%	(*n* = 3)	10.61%	(*n* = 7)
Some college credit, but no degree	32.61%	(*n* = 30)	30.30%	(*n* = 20)
Associate's degree (AA or AS)	17.39%	(*n* = 16)	13.64%	(*n* = 9)
Bachelor's degree (BA or BS)	9.78%	(*n* = 9)	18.18%	(*n* = 12)
Graduate or professional degree	9.78%	(*n* = 9)	7.58%	(*n* = 5)
**Individual total annual income, % (*****n*****)**	3.26%	(*n* = 3)	3.03%	(*n* = 2)
Missing				
<$10,000	34.78%	(*n* = 32)	28.79%	(*n* = 19)
$10,000–$19,999	21.74%	(*n* = 20)	9.09%	(*n* = 6)
$20,000–$29,999	8.70%	(*n* = 8)	21.21%	(*n* = 14)
$30,000–$39,999	9.78%	(*n* = 9)	13.64%	(*n* = 9)
$40,000–$49,999	11.96%	(*n* = 11)	15.15%	(*n* = 10)
$50,000–$59,999	4.35%	(*n* = 4)	3.03%	(*n* = 2)
$60,000–$79,999	4.35%	(*n* = 4)	3.03%	(*n* = 2)
$80,000 or more	1.09%	(*n* = 1)	3.03%	(*n* = 2)
**Household total annual income, % (*****n*****)**	11.96%	(*n* = 11)	4.55%	(*n* = 3)
Refused/Unknown/Missing				
<$10,000	1.09%	(*n* = 1)	3.03%	(*n* = 2)
$10,000–$19,999	8.70%	(*n* = 8)	3.03%	(*n* = 2)
$20,000–$29,999	7.61%	(*n* = 7)	10.61%	(*n* = 7)
$30,000–$39,999	15.22%	(*n* = 14)	22.73%	(*n* = 15)
$40,000–$49,999	11.96%	(*n* = 11)	15.15%	(*n* = 10)
$50,000–$59,999	15.22%	(*n* = 14)	9.09%	(*n* = 6)
$60,000–$79,999	20.65%	(*n* = 19)	18.18%	(*n* = 12)
$80,000 or more	7.61%	(*n* = 7)	13.64%	(*n* = 9)
**Insurance^**a**^, % (*n*)**	1.09%	(*n* = 1)	0%	(*n* = 0)
Missing				
Private Insurance, through employer	21.74%	(*n* = 20)	33.33%	(*n* = 22)
Private Insurance, through private insurer	5.43%	(*n* = 5)	4.55%	(*n* = 3)
Private Insurance, through marketplace	4.35%	(*n* = 4)	0%	(*n* = 0)
Medicare	6.52%	(*n* = 6)	3.03%	(*n* = 2)
MediGap	0%	(*n* = 0)	3.03%	(*n* = 2)
Medicare Part D	2.17%	(*n* = 2)	1.52%	(*n* = 1)
Medicaid	7.61%	(*n* = 7)	4.55%	(*n* = 3)
CHAMP VA, not from CSP^b^	3.26%	(*n* = 3)	6.06%	(*n* = 4)
CHAMP VA, from CSP	0%	(*n* = 0)	0%	(*n* = 0)
TRICARE	41.30%	(*n* = 38)	43.94%	(*n* = 29)
VA	0%	(*n* = 0)	1.52%	(*n* = 1)
Indian Health Service	0%	(*n* = 0)	0%	(*n* = 0)
Other	3.26%	(*n* = 3)	7.58%	(*n* = 5)
No Health Insurance	11.96%	(*n* = 11)	7.58%	(*n* = 5)
**Employment status after becoming a caregiver, % (*****n*****)**	2.17%	(*n* = 2)	0%	(*n* = 0)
Missing				
I am working my usual hours for pay	22.83%	(*n* = 21)	27.27%	(*n* = 18)
I am working reduced hours for pay	21.74%	(*n* = 20)	28.79%	(*n* = 19)
I started working for pay	1.09%	(*n* = 1)	0%	(*n* = 0)
I started working more hours for pay	3.26%	(*n* = 3)	0%	(*n* = 0)
I stopped working for pay completely	34.78%	(*n* = 32)	28.79%	(*n* = 19)
I was not working before and am not now	14.13%	(*n* = 13)	15.15%	(*n* = 10)

a *Categories are not mutually exclusive. Multiple responses allowed*.

b *Caregiver support program. Table displays descriptive statistics for the final study cohort (n = 158)*.

Ninety-six percent of caregivers in the participating group and 89% of caregivers in the non-participating group lived in the same house as the Veteran. This high rate is a result of the policy requirement: if a non-related individual receives the benefit they must live in the same household. Around 30% of participating and non-participating caregivers had completed some college but not obtained a degree; and ~37% in both groups reported obtaining an Associate's Degree or higher. Similar proportions of participating and non-participating caregivers reported having health care insurance through Tricare, the most commonly reported health insurance. Finally, over half of caregivers reported working reduced hours or stopping work completely since becoming a caregiver (57%).

### PCAFC and Caregiver Well-Being Outcomes

Table 2 displays the descriptive statistics for the well-being measures of the 158 caregivers included in the analysis. For some measures, the number of available responses was lower due to missing or incomplete subscales.

**Table 2:** Caregiver well-being descriptive results^a^.

	**Analytic cohort**							
	**Participants**				**Non-participants**			
	**Survey 1**		**Survey 2**		**Survey 1**		**Survey 2**	
Caregiver financial strain^b^, mean (*SD*)	3.64 (0.97)	(*n* = 81)	3.28 (1.17)	(*n* = 81)	3.83 (0.84)	(*n* = 60)	3.84 (0.95)	(*n* = 60)
Caregiver depressive symptoms^c^, mean (*SD*)	8.84 (6.18)	(*n* = 85)	8.56 (6.37)	(*n* = 85)	10.28 (6.90)	(*n* = 50)	11.65 (7.53)	(*n* = 50)
Caregiver perceived quality of VA care, mean^d^ (*SD*)	6.21 (2.75)	(*n* = 76)	5.96 (2.89)	(*n* = 76)	6.10 (2.55)	(*n* = 42)	5.29 (2.80)	(*n* = 42)
Caregiver burden: zarit scale^e^, mean (*SD*)	16.88 (10.19)	(*n* = 85)	15.85 (10.76)	(*n* = 85)	18.17 (11.38)	(*n* = 53)	21.43 (12.48)	(*n* = 53)
Positive aspects of caregiving scale^f^, mean (*SD*)	34.26 (8.83)	(*n* = 85)	35.51 (8.70)	(*n* = 85)	33.30 (8.99)	(*n* = 54)	32.87 (10.11)	(*n* = 54)

a *Results presented for each item reflect responses from individuals who had scores at both survey time points*.

b *Financial strain score range 1 (low strain)-5 (high strain)*.

c *CESD scale range 0 (low depressive symptoms)-30 (high depressive symptoms)*.

d *Perceived quality of care scale range 0 (worst care)-10 (best care)*.

e *Zarit scale range 0 (positive perspective)-48 (very stressed)*.

f *Positive Aspects of Caregiving Scale range 9 (very negative)-45 (very positive)*.

*Table displays descriptive statistics for the well-being measures of the 158 caregivers that were included in analysis. For some measures, the number of available responses was lower due to missing or incomplete subscales*.

#### Perceived Financial Strain

**Figure 2:**
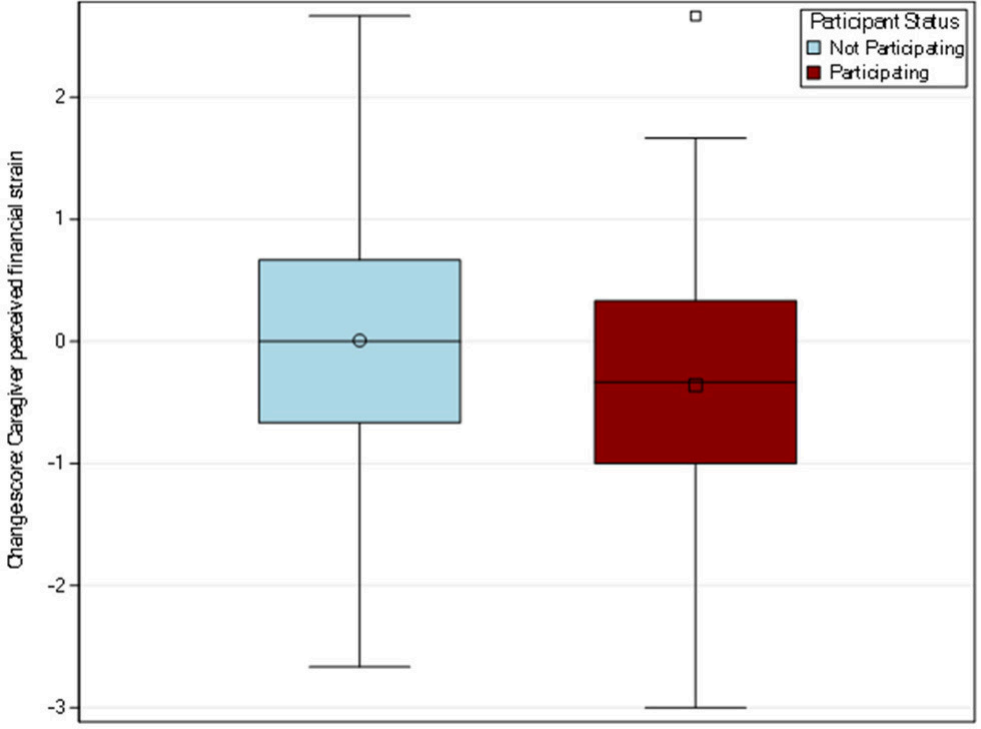
Distribution of change scores for caregiver financial strain by participation. Perceived financial strain was measured through the three-item impact on finances subscale from the caregiver reaction assessment. The participating caregiver group had a mean change of −0.36 while the non-participating caregiver group had a mean change of 0.008.

The participating caregiver group mean score changed from 3.64 to 3.28 for a mean change of −0.36. The non-participating caregiver group mean score changed from 3.83 to 3.84 for a mean change of 0.008 (see Figure 2). The t-test provided moderate evidence for a statistically significant difference in change between the participating and non-participating groups (*p* = 0.04).

#### Caregiver Depressive Symptoms (CESD-10)

**Figure 3:**
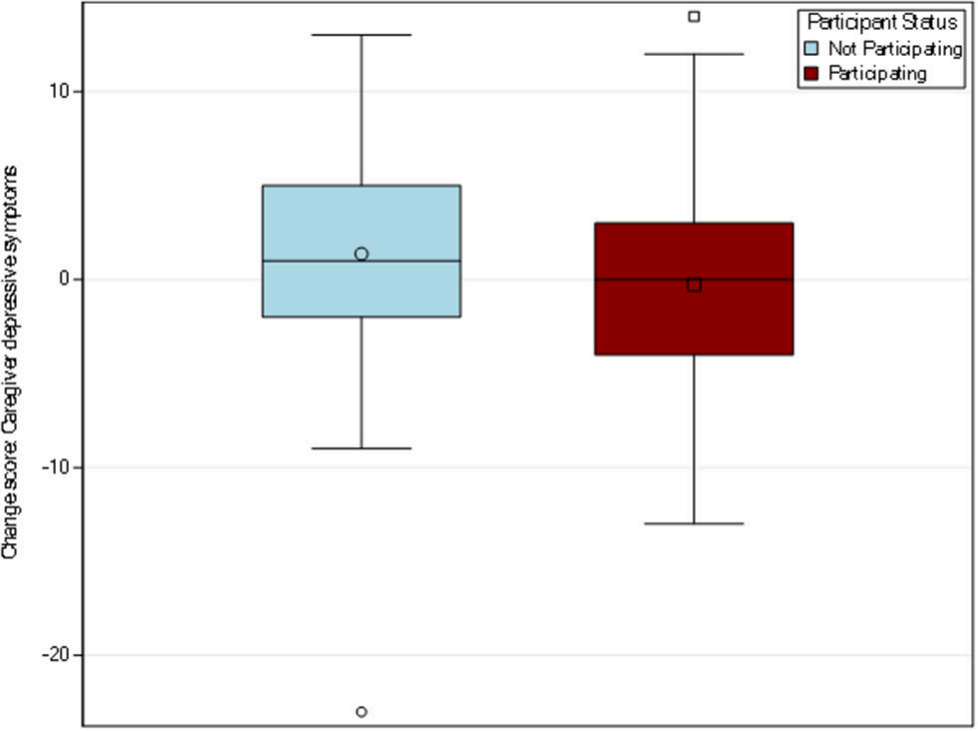
Distribution of change scores for caregiver depressive symptoms by participation. Out of the resulting sample size, *n* = 158 respondents, 135 caregivers completed the CESD-10 in both pre-post-surveys. The participating caregiver group had a mean change of −0.27 while the non-participating caregiver group had a mean change of 1.37.

Among the 135 caregivers who completed the CESD-10 in both surveys, the participating caregiver group's mean score dropped from 8.84 to 8.56, for a mean change of −0.27. For the non-participating group, the mean score increased from 10.28 to 11.65, a mean change of 1.37 (see Figure 3). The *t*-test did not indicate a statistically significant difference between the participating and non-participating groups (*p* = 0.11). Upon examining the distribution of the CESD-10 change score, one particularly influential respondent was identified whose change in CESD-10 was vastly greater than the remainder of the caregivers in the sample. Specifically, the outlying value indicated a 9 month change that was nearly as extreme as possible and was almost 5 standard deviations away from the group mean. As sensitivity analyses, both a *t*-test was rerun with the influential individual removed and a Wilcoxon rank sum test, a non-parametric analog to the *t*-test, was conducted on the full *n* = 135 sample. With the outlier individual removed, the mean difference in CESD-10 score in the non-participating group increased to 1.86 and the *t*-test indicated statistical significance (*p* = 0.02). The Wilcoxon rank sum test provided a *p*-value of 0.09. The variations in estimates and statistical significance reiterate the need to interpret results from this analysis as preliminary and meriting future work.

#### Caregiver Perception of VA Quality of Care

**Figure 4:**
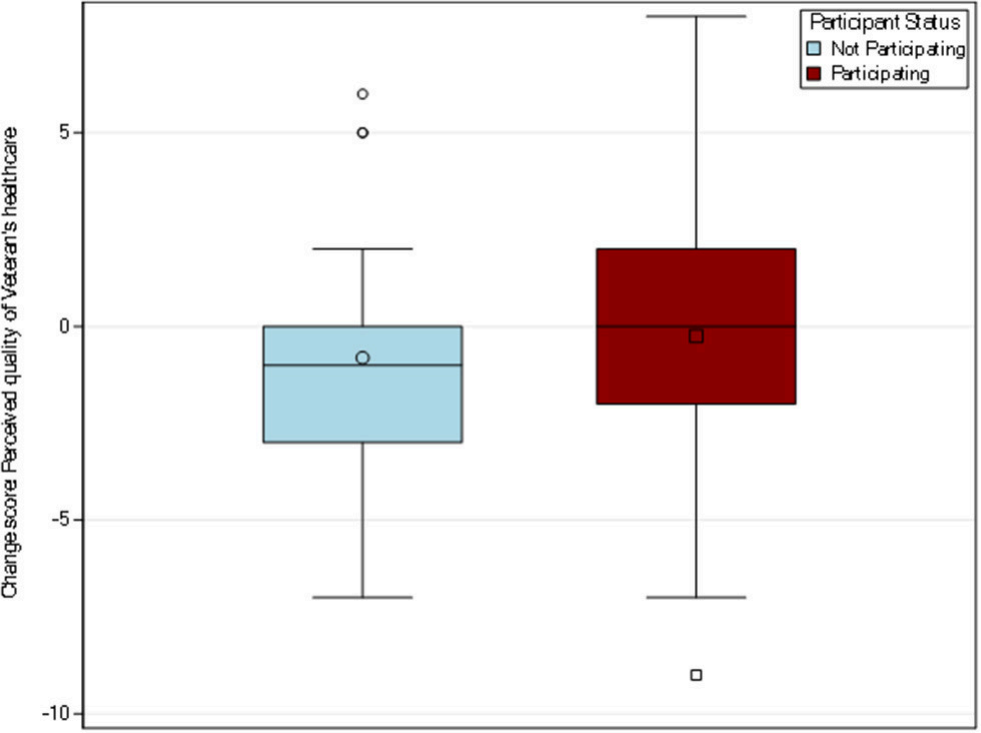
Distribution of change scores for caregiver perception of quality of VA care. A single item from the CAHPS 2013 Health Plan survey was used to measure caregiver's global rating of satisfaction with the Veteran's VHA care. Among the 118 caregivers who responded to this item, the participating caregiver group mean change was −0.25 while the non-participating caregiver group mean change was −0.81.

Among the 118 caregivers who responded to this item, the participating caregiver group mean rating changed from 6.21 to 5.96, for a mean change of −0.25, and the non-participating group mean rating changed from 6.10 to 5.29 for a mean change of −0.81 (see Figure 4). A *t*-test suggested no difference between the groups (*p* = 0.33). Results may change with a larger and more generalizable sample, but compared to the other outcomes (perceived financial strain and depressive symptoms), VA quality of care ratings from the caregiver perspective may not be as meaningful an outcome for future inquiry.

##### Caregiver burden

**Figure 5:**
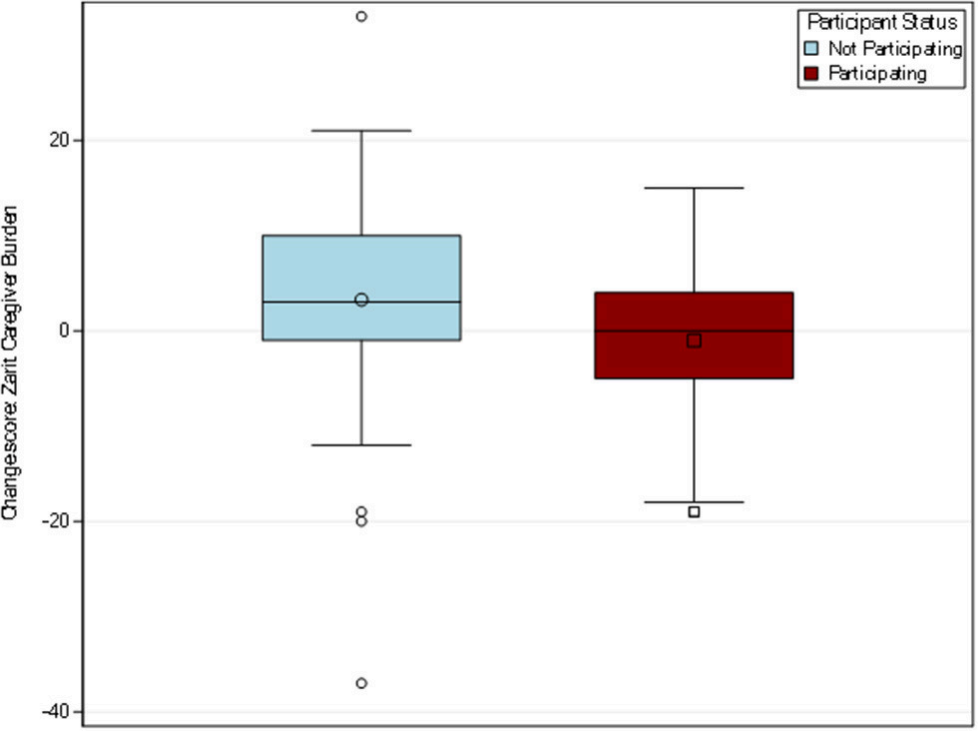
Distribution of change scores for caregiver burden. Out of the resulting sample size (*n* = 158), 138 caregivers responded to the 12-item Zarit burden measure to describe the level of stress felt by a caregiver. The participating caregiver group had a change of 1.04 while the non-participating caregiver group had a mean change of 3.26.

Among the 138 caregivers who responded to this item at both time points, the participating caregiver group mean score decreased from 16.88 to 15.85, for a mean change of −1.04. The non-participating group mean score increased from 18.17 to 21.43, for a mean change of 3.26 (see Figure 5). The *t*-test indicated a statistically significant difference between the participating and non-participating groups (*p* = 0.01). These results suggest promise of a positive trend in caregiver burden among those participating in PCAFC and should be examined further in future studies.

##### Positive aspects of caregiving

**Figure 6:**
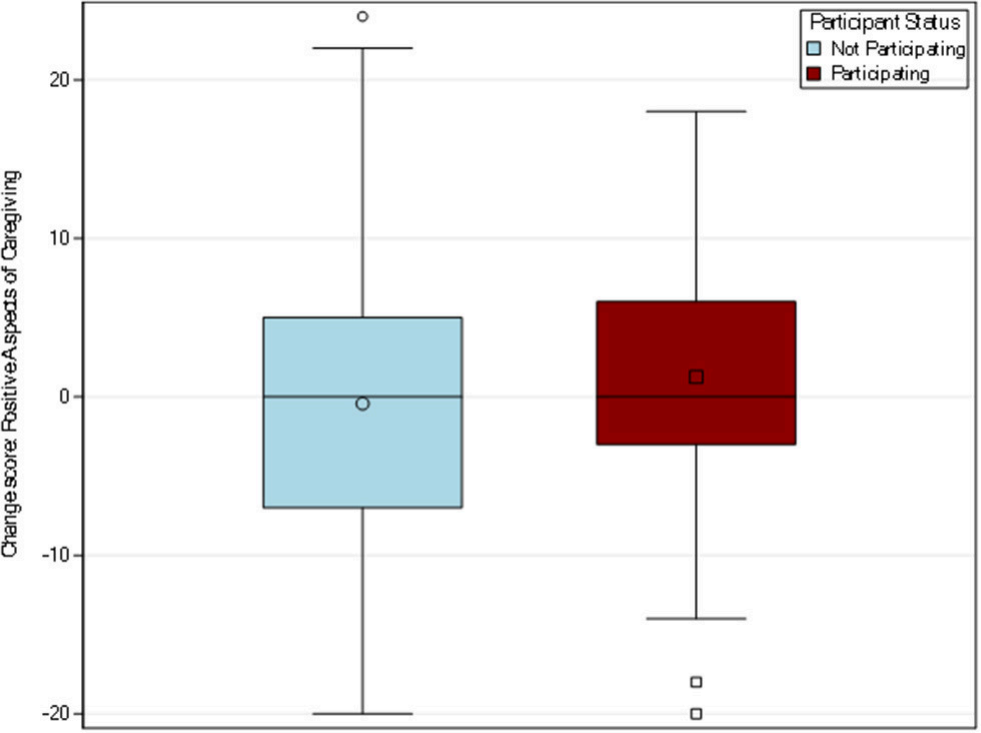
Distribution of change scores for positive aspects of caregiving. Positive aspects of caregiving was measured using a well-validated measure identified by Tarlow et al. (23). Among the 139 caregivers responding to this item in both pre and post-surveys, the participating caregiver group mean change was 1.25 while the non-participating caregiver group had a mean change of −0.43.

Among the 139 caregivers responding to this item at both time points, the participating caregiver group mean score increased from 34.26 to 35.51, for a mean change of 1.25, while the mean score for non-participants decreased from 33.30 to 32.87, for a mean change in −0.43 (see Figure 6). The *t*-test did not indicate a statistically significant difference between the participating and non-participating groups (*p* = 0.24).

## Discussion

There is a dearth of knowledge about how caregiver well-being responds to comprehensive caregiver support in the U.S., primarily because comprehensive support programs have been nearly non-existent prior to the 2010 law establishing the PCAFC. As such, this study provides a first glimpse of potential trends in caregiver well-being outcomes for those who receive comprehensive support. Whereas, it may seem obvious at first glance that a program that includes a monthly stipend of $600–$2,300 on average should reduce perceived financial strain, given commensurate work reductions, it was not clear to us that it would from the outset. For some dyads, the stipend may not have replaced the lost earnings that they experienced since the Veteran's injury whereas for others the stipend may have been a new source of financial support (for example, if the caregiver was not working prior to the Veteran's injury). Among caregivers, leaving employment or reducing hours in both groups are high; further across both groups the Veteran care recipient may also be less likely to work. Therefore, financial strain was likely to be high for dyads who applied to the program despite existing pensions or disability payments directly received by the Veteran, making it unclear *a priori* whether the PCAFC's monthly stipend would be sufficient to reduce perceived financial strain for participants compared to non-participants. We found that the perception of financial strain decreased among participants, but due to the low response, this relationship warrants further study. While there is no accepted metric of meaningful change in the financial strain scale, our observed change of 0.37 is in line with changes observed in other studies (14, 15). However, it is unclear how this translates to the caregivers' perspective of how meaningfully this difference was felt. Second, whereas depressive symptoms did not improve for the PCAFC caregivers in the primary analysis, there was a promising trend toward differences in depressive symptoms across groups, particularly after removing an extreme outlying response. In fact, depressive symptoms appeared to worsen in non-participants and remain the same among participants. This increase could be in part due to exacerbated financial strain and lack of supports from the program or lack of medical care access for the caregiver's Veteran compared to participants (24), but more study is needed among larger samples. Training for participants may have increased self-efficacy and/or coping thereby preventing an increase in depressive symptoms.

Despite no observed differences in quality of care, overall responses were lower than those observed in other VA studies (25, 26), largely surveying older Veterans regarding their own care. However, there is a dearth of literature on overall VA satisfaction measurements provided by younger caregivers, with one recent study showing that 75% of caregivers were mostly or very satisfied with their Veteran's inpatient care at VA (25).

The survey's demographics and less-commonly examined measures of caregiver experience, such as positive aspects of caregiving and perceived financial strain, help provide contextual information about this unique and understudied group of U.S. caregivers. Retaining classic caregiver measures, such as Zarit burden and CESD10 for strain and depressive symptoms, respectively, allows comparability with other caregiver studies. Specifically, Zarit burden decreased among participating caregivers and increased among non-participating caregivers at levels that are comparable to those found in randomized control trials that provide psycho-educational support and/or training to family caregivers of older adults (e.g., 2 points). This preliminary finding merits additional inquiry in a study that allows better control for potential confounders and in a more generalizable sample.

Additionally, caregivers of post-9/11 Veterans are juggling different sets of responsibilities (e.g., post-secondary education choices, fertility decisions, and/or rearing of young children) when they assume the caregiver role, compared to persons who become caregivers of older dependent adults who have experienced a loss of independence. Moreover, the caregivers of post-9/11 Veterans are often providing different types of care than traditional caregivers of older dependent adults, such as a focus on managing mental health illnesses and injuries (1). Our sample of post-9/11 caregivers, those participating in PCAFC and those not participating, reported providing more continual care than in the RAND study, with nearly 7 days of full-time care being provided a week on average. However, our sample is similar to the RAND military caregiver sample on education characteristics, there are critical differences in other observed demographic characteristics (1). Our sample of caregivers included a much larger percentage of female respondents (>90% compared to RAND's 60%), more spouses/significant others (>85% compared to RAND's 33% of spouses), and fewer parents (<10% compared to RAND's 25%). Our sample may be caring for a more severely injured cohort of Veterans compared to the RAND study, but making strong conclusions about this is not possible without knowing what proportion of the RAND sample, which was surveyed in August-October 2013, were later enrolled in the PCAFC.

The PCAFC program is the most comprehensive family caregiver program ever enacted in the U.S. While it is estimated to have cost $1 billion as of May 2015, and is expected to grow to around $450 million per year, PCAFC still touches very few family caregivers of the total military caregiving population, because of the narrow eligibility criteria in the law. Of 1.1 million post-9/11 military caregivers, 33,000 (or 3% of the total post-9/11 caregiving population) to date have participated in PCAFC in the past 6 years since its inception. Many of these 1.1 million post-9/11 caregivers provide care for Veterans who have lower functional impairment or have functional impairments that are from illness not injury, making them ineligible for PCAFC. The total number of caregivers of Veterans from all eras is estimated to be 5.5 million (1), thus PCAFC participants represent only 0.6% of all caregivers of Veterans.

As stated previously, there are significant limitations to this analysis. First, this analysis has a very small sample size. There was an extremely low response rate, particularly when considering caregivers who responded to both surveys, such that our analysis represents <5% of caregivers invited to participate in the surveys and precluded the originally planned rigorous comparative effectiveness design. Second, due to the regulatory difficulties in getting approval for a web-based survey, Survey 1 and Survey 2 used different modes of data collection. Third, this analysis is subject to self-selection bias of those who returned surveys compared to those who did not. Lastly, those denied enrollment could be inherently different compared to those enrolled in ways we did not capture. While we lack specific information on the reasons for denial of participation, most commonly they were denied due to administrative reasons, such as being from an earlier service era (pre-9/11) or requiring a caregiver due to illness not injury (law requires it be injury). These limitations preclude over-generalization of the results.

Given the limitations above, this analysis shows promising trends for future evaluation. Almost all of the outcomes descriptively suggested a positive programmatic influence, with some being close to or reaching statistical significance. With a larger, more representative sample, it is possible differences would be detected in more of the outcomes. However, it is also possible that differences are due to selection bias and thus would attenuate with a more representative sample. Future inquiries should use mixed methods to delve into the differential effects of different services and programs within VA CSP on caregiver well-being outcomes. We know that uptake of the optional services and programs in PCAFC are relatively low (11), hence we believe that the effects found in this paper can be explained by the components common to all participants, the monthly stipend and the mandatory training. These preliminary study results reinforce the need for future research to examine these outcomes further with a larger, more representative sample. This future study should also be designed to enable us to quantify the level of interdependence between financial strain and emotional strain, to effectively target resources toward ameliorating caregivers' unmet needs and in turn improve quality of care that they can provide.

## Ethics Statement

As a VA quality improvement project, this work was not subject to Institutional Review Board (IRB) approval.

## Author Contributions

VS assisted with developing study design, analyzed, and interpreted results and was a major contributor in writing of the manuscript. JL analyzed and interpreted results and was a major contributor to the writing of the manuscript. KM and MS-B contributed to the interpretation of results and writing of the manuscript and analyses. MO provided guidance for the study design, analysis, interpreted results, and was a major contributor in writing of the manuscript. MC-K, JH, and MK all contributed to study design and contributed to the manuscript. CV contributed to the study design, analysis plan, and interpretation of results and was a major contributor to the manuscript. All authors read and approved the final manuscript.

### Conflict of Interest Statement

While, the authors declare no conflicts of interest, as stated in the cover letter and Acknowledgments section, this research was funded by the VA Caregiver Support Program as part of a broader evaluation of the program on caregiver and Veteran health and health service use outcomes. The funding partners are co-authors (MK, JH, and MC-K) and have reviewed and approved this paper. However, the funders were not involved in the analysis and interpretation of the data. As authors, the funders provided information about the structure of the program to inform the evaluation design, they reviewed drafts of the paper, and have given their approval for it to be published.

## References

[1] Ramchand R Tanielian T Fisher MP Vaughan CA Trail TE Epley C. Hidden heroes: America's Military Caregivers. Rand Health. (2014) 4:14. 10.7249/RR499 28083343 PMC5052006

[2] Levine C Hunt GG Halper D Hart AY Lautz J Gould DA. Young adult caregivers: a first look at an unstudied population. Am J Public Health. (2005) 95:2071–75. 10.2105/AJPH.2005.067702 16195506 PMC1449485

[3] Schulz R Beach SR Lind B Martire LM Zdaniuk B Hirsch C. Involvement in caregiving and adjustment to death of a spouse: findings from the caregiver health effects study. J Am Med Assoc. (2001) 285:3123–9. 10.1001/jama.285.24.3123 11427141

[4] Coe NB Van Houtven CH. Caring for mom and neglecting yourself? The health effects of caring for an elderly parent. Health Econ. (2009) 18:991–1010. 10.1002/hec.1512 19582755

[5] Van Houtven CH Friedemann-Sánchez G Clothier B Levison D Taylor BC Jensen AC Is policy well-targeted to remedy financial strain among caregivers of severely injured U.S. Service Members? Working Paper. Durham: Durham VA HSR&D: VAMC (2011). 10.5034/inquiryjrnl_49.04.01 23469677

[6] Van Houtven CH Friedemann-Sánchez G Clothier B Levison D Taylor BC Jensen AC Is policy well-targeted to remedy financial strain among caregivers of severely injured U.S. Service Members? Inquiry. (2012) 49:339–51. 10.5034/inquiryjrnl_49.04.01 23469677

[7] Griffin JM Lee MK Bangerter LR Van Houtven CH Friedemann-Sánchez G Phelan SM. Burden and mental health among caregivers of veterans with traumatic brain injury/polytrauma. Am J Orthopsychiatry. (2017) 87:139–48. 10.1037/ort0000207 28206801

[8] Malec J Van Houtven CH Tanelian T Doran M Impact of TBI on caregivers of veterans with TBI: a review of burden and interventions. Brain Inj. (2016) 31:1235–45. 10.1080/02699052.2016.1274778 28981343

[9] Patel BR. Caregivers of veterans with invisible injuries: what we know and implications for social work practice. Social Work. (2015) 60:9–17. 10.1093/sw/swu043 25643571

[10] Stevens LF Pickett TC Wilder Schaaf KP Taylor BC Gravely A Van Houtven CH. The relationship between training and mental health among caregivers of individuals with polytrauma. Behav Neurol. (2015) 2015:185941. 10.1155/2015/185941 26770015 PMC4685074

[11] Sperber N Van Houtven C Andrews S Miller K Steinhauser KE Smith V Family caregiver use and value of support services in the VA Program of comprehensive assistance for family caregivers. J Long Term Care. (2018) 41–50. 10.21953/lse.n3tazsz5zmai

[12] Miller KEM Lindquist JH Olsen MK Smith V Voils CI Oddone EZ. Invisible partners in care: snapshot of well-being among caregivers receiving comprehensive support from Veterans Affairs. Health Sci Rep. (2019) 2:e112. 10.1002/hsr2.112 30937391 PMC6427058

[13] Given CW Given B Stommel M Collins C King S Franklin S. The caregiver reaction assessment (CRA) for caregivers to persons with chronic physical and mental impairments. Res Nurs Health. (1992) 15:271–83. 10.1002/nur.4770150406 1386680

[14] Nightingale CL Curbow BA Wingard JR Pereira DB Carnaby GD. Burden, quality of life, and social support in caregivers of patients undergoing radiotherapy for head and neck cancer: a pilot study. Chronic Illn. (2016) 12:236–45. 10.1177/1742395316644305 27068111 PMC5515480

[15] Nijboer C van den Bos GAM Triemstra M Mulder M Tempelaar R Sanderman R. Patterns of caregiver experiences among partners of cancer patients. Gerontologist. (2000) 40:738–46. 10.1093/geront/40.6.738 11131090

[16] Andresen EM Malmgren JA Carter WB Patrick DL. Screening for depression in well older adults: evaluation of a short form of the CES-D. Am J Prev Med. (1994) 10:77–84. 10.1016/S0749-3797(18)30622-6 8037935

[17] Hargraves JL Hays RD Cleary PD. Psychometric properties of the Consumer Assessment of Health Plans Study (CAHPS) 2.0 adult core survey. Health Serv Res. (2003) 38:1509–27. 10.1111/j.1475-6773.2003.00190.x 14727785 PMC1360961

[18] Bedard M Molloy DW Squire L Dubois S Lever JA O'Donnell M. The Zarit Burden Interview: a new short version and screening version. Gerontologist. (2001) 41:652–7. 10.1093/geront/41.5.652 11574710

[19] Zarit SH Reever KE Bach-Peterson J. Relatives of the impaired elderly: correlates of feelings of burden. Gerontologist. (1980) 20:649–55. 10.1093/geront/20.6.649 7203086

[20] Schulz R O'Brien A Czaja S Ory M Norris R Martire LM. Dementia caregiver intervention research: in search of clinical significance. Gerontologist. (2002) 42:589–602. 10.1093/geront/42.5.589 12351794 PMC2579772

[21] Sorensen S Pinquart M Duberstein P. How effective are interventions with caregivers? An updated meta-analysis. Gerontologist. (2002) 42:356–72. 10.1093/geront/42.3.356 12040138

[22] O'Rourke N Tuokko HA. Psychometric properties of an abridged version of The Zarit Burden Interview within a representative Canadian caregiver sample. Gerontologist. (2003) 43:121–7. 10.1093/geront/43.1.121 12604753

[23] Tarlow B Wisniewski S Belle S Rupert M Ory M Gallagher-Thompson D Positive aspects of caregiving: contributions of the REACH project to the development of new measures for Alzheimer's research. Res Aging. (2004) 26:429–53. 10.1177/0164027504264493

[24] Van Houtven CH Smith VA Stechuchak KM Shepherd-Banigan M Hastings SN Maciejewski ML Comprehensive support for family caregivers: impact on veteran health care utilization and costs. Med Care Res Rev. (2017) 76:89–114. 10.1177/1077558717697015 29148338 PMC5726944

[25] Nelson KM Helfrich C Sun H Hebert PL Liu CF Dolan E. Implementation of the patient-centered medical home in the Veterans Health Administration: associations with patient satisfaction, quality of care, staff burnout, and hospital and emergency department use. JAMA Intern Med. (2014) 174:1350–8. 10.1001/jamainternmed.2014.2488 25055197

[26] Veterans Affairs American Customer Satisfaction Index: 2013 Customer Satisfaction Outpatient Survey. Washington, DC: Veterans Affairs (2013).

